# Different ways to make neurons: parallel evolution in the *SoxB* family

**DOI:** 10.1186/gb4177

**Published:** 2014-05-30

**Authors:** Nathalie Neriec, Claude Desplan

**Affiliations:** 1 Department of Biology, New York University, 1009 Silver Center, 100 Washington Square East, NY 10003-6688, USA; 2 Center for Genomics & Systems Biology, New York University Abu Dhabi Institute, Abu Dhabi, UAE

**Keywords:** Neuronal development, *SoxB*, Functional conservation, *Drosophila*

## Abstract

Combining genome-wide analyses of binding sites and expression profiles generates a model for the functional evolution of two SOXB paralogous proteins in neurogenesis.

## 

How conserved are neuronal developmental programs between vertebrates and invertebrates? Although this remains a major open question, breakthroughs have recently been made in understanding the genetic networks involved in neuronal development, including neuronal specification, stem cell maintenance and neural fate commitment. In parallel, technological advances in sequencing have improved our understanding of evolutionary events in protein families. However, it has not yet been possible to determine how the evolution of individual genes has affected the function of networks during neuronal development.

In a recent study, Ferrero *et al*. [1] use a combination of genome-wide transcription factor binding and expression profiling to investigate the evolution of the *SoxB* family of genes involved in neuronal development.

## The *SoxB* family in neuronal development 

**Figure 1:**
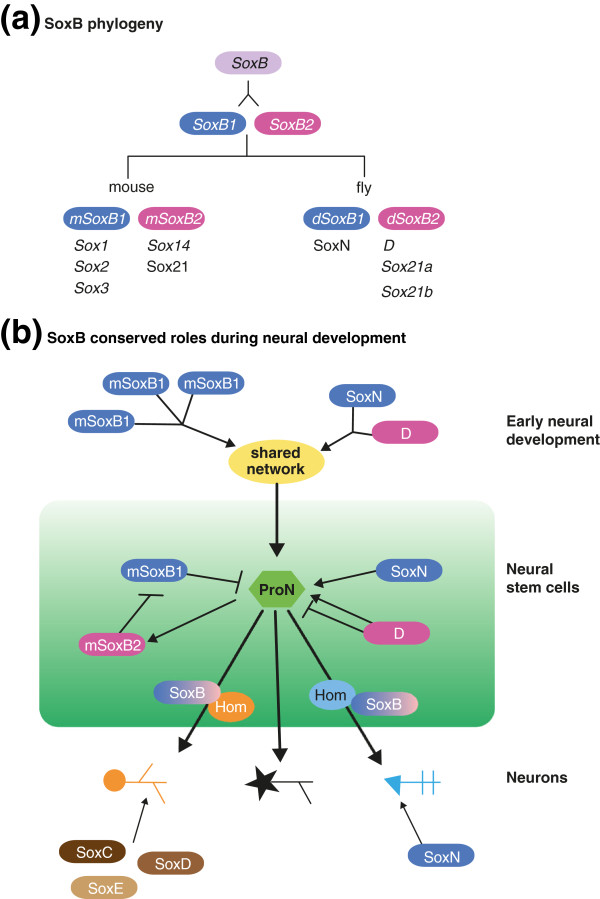
**Evolution of the SoxB family. (a)** A duplication event in the *SoxB* family generated an ancestral SoxB1 and a SoxB2 before the split between invertebrates and vertebrates (adapted from Zhong *et al*. [3]). **(b)** The SoxB proteins are involved in three main developmental steps of the nervous system: in very early neural development to generate stem cells, in the maintenance of stem cells, and later in post-mitotic differentiating neurons.

*SoxB* genes encode HMG transcription factors (SOXBs) that are essential for neuronal development in a range of model organisms, including sea urchins, flies, amphibians and mice [2]. The highly conserved *SoxB* family can be divided into two main subgroups, *SoxB1* and *SoxB2*. The duplication event that led to their formation occurred before the evolutionary split between vertebrates and invertebrates (Figure 1a), and both the *SoxB1* and *SoxB2* families exist in both phyla [3].

In mammals, the *SoxB1* family contains three genes, *Sox1*, *Sox2* and *Sox3*, and the *SoxB2* family contains two genes, *Sox14* and *Sox21*. All mammalian SOXB1 proteins (mSOXB1) are redundant transcriptional activators with very similar patterns of expression during early establishment of the neurectoderm (Figure 1b). In later steps, SOXBs are essential for the maintenance of neural stem cells, and *SoxB1* genes are repressed by the mSOXB2 repressor proteins once neural stem cells start expressing proneural genes and are committing to neural fate [2]. In *Drosophila*, the distinction between *SoxB1* and *SoxB2* is less well understood. The fly genome contains four *SoxB* genes, but only two are expressed in the developing embryonic nervous system: SoxNeuro (*SoxN*), which belongs to the SoxB1 group, and Dichaete (*D*), which is closer to the *SoxB2* family. In the embryo, both *SoxN* and *D* are expressed and required to control the establishment of neural stem cells and, later, they control the proneural genes *achaete scute* and *asense* in neural stem cells called neuroblasts in flies [4].

What were the ancestral functions of SOXBs and how did they evolve in parallel between vertebrates and invertebrates? In order to address this question, the recent studies from Ferrero *et al*. [1] and Aleksic *et al*. [5] identify the genomic targets of both SoxN and D proteins and investigate their ability to compensate for the loss of each other with *in vivo* binding assays. These studies provide a profound insight into the roles and redundancies of *SoxN* and *D*, and insights into the possible functional conservation of SOXB proteins in three major aspects of nervous system development.

## Three ancestral roles for *SoxBs*

### A major early regulatory hub

Both mSOXB1s and SoxN/D control the early activation of a robust and highly conserved network required to set up the nervous system in the embryo. Only triple mutants for m*SOXB1s* or double mutants for *D* and *SoxN* show severe neural hypoplasia, failing to form neural stem cells [2,4]. Furthermore, *Sox2* can rescue the lack of *D* if expressed early in development [4]. By performing genome-wide analyses of binding, Ferrero *et al*. [1] show that *SoxN* and *D* are early transcriptional activators of a shared core set of targets enriched for transcription factors and effectors with roles in neural development. In mammals, duplication of the ancestral *SoxB1* has increased the robustness of SOXB1s as an early activator, while the paralogous *SoxB2* genes have acquired new functions as repressors. However, in *Drosophila*, whose genome contains only one *SoxB1* (*SoxN*), *D* (*SoxB2*) has remained highly redundant with *SoxN* to maintain robustness. This highlights the fact that paralogous genes may have been selected for redundancy to maintain the robustness of essential developmental networks.

### Function in neural stem cells

To generate post-mitotic neurons at a later stage in development, neural stem cells must repress stem cell-like features and express pro-neural genes, which control the commitment of the cell to a neural fate. In mammals, *SoxB1s* repress proneural genes and *SoxB2s* promote neural fate. In *Drosophila*, *SoxN* (*SoxB1*) promotes expression of proneural genes *achaete scute* and *asense*, and *D* (*SoxB2*) represses them in the intermediate column of the developing nervous system. In both of those cases, SOXB proteins have maintained opposite functions in the regulation of proneural genes to promote neural fate. In another part of the developing *Drosophila* nervous system called the medial column, both D and SOXN activate the expression of achaete scute, leading to the transition from stem cell state to neuron [4]. At a more general level, Ferrero *et al*. [1] show that most if not all proneural genes are direct targets of SOXN and D. This indicates that ancestral SOXB proteins most likely played roles in the production of neurons by controlling the balance between stem cell maintenance and differentiation. Whether these ancestral roles were through antagonistic functions remains unclear.

The development of the nervous system requires not only the generation of post-mitotic neurons, but also the production of different types of neurons with very specific properties based on their spatial position and birthdate. In both vertebrate neural tube precursors and *Drosophila* embryonic ventral nerve cord neuroblasts, a set of homeodomain transcription factors are co-expressed with *SoxBs* to control the patterning of different types of neural stem cells. This suggests that an interaction between homeoproteins and SOXBs has been conserved across evolution to pattern the spatial identity of neural stem cells during early development. In flies, both *SoxN* and *D* also seem to be redundantly involved in the integration of patterning information [1]. More studies are needed to understand the involvement of *SoxB1s* and SoxB2 in patterning the mammalian central nervous system.

Interestingly, the functions of SOXB proteins to balance self renewal versus commitment to neural fate and the integration of patterning information to establish proper neuronal identities occur around the same time, most likely in the same cells. How these genes can have two parallel functions remains unknown, although this may take advantage of the fact that SOXBs functional specificity is highly dependent on binding partner.

### Later stage of neuronal differentiation

SOX proteins are known to be involved in later stages of neuronal differentiation. However, in mammals, other groups of SOX proteins, SOXC, SOXD and SOXE, play roles in terminal differentiation of neurons. It is possible that SOXB proteins have a later role that remains to be discovered [6]. In flies, the study from Ferrero *et al*. [1] identified a set of SOXN-specific targets that are involved in terminal differentiation. However, binding to those targets is lost when *D* is mutated, suggesting that SOXN and D work together to activate these genes. This type of late role and interdependency between SOXN and D was unexpected. This could be similar to mammals, where SOXD and SOXE have been shown to heteromerize in order to bind to their targets to control terminal differentiation. However, understanding the epistatic relationship between *SoxN* and *D* will be necessary for eliminating the possibility that loss of SOXN binding is due to indirect effects of the loss of D earlier in the process.

## Perspectives

Ferrero *et al*. [1], along with other recent studies, have shed light on the possible roles that original *SoxB* genes played, and how the mammalian and fly *SoxB* genes have evolved independently. Early in neural development, SOXB proteins are redundant and the main regulators of a well-conserved early transcriptional network. Later in stem cells, they play a role in the regulation of proneural genes to control the exit of a stem cell state; they also interact with homeodomain proteins to control neuronal identities. How these functions and interactions evolved in parallel with other pathways and how similar or divergent they are today remain questions to be answered.

Interestingly, although *SoxB* genes have similar functions in mammals and flies, evolution led to a different division of labor between B1 and B2 paralogs. While SOXB1s and SOXB2s represent two highly specialized and distinct subfamilies of proteins in mammals, fly SOXN and D have maintained a high level of redundancy in neural development with a set of unique targets. Interestingly, *SOXN* has the ability to compensate for D, even at D-specific sites, while D does not compensate as much for the loss of *SoxN*: some SOXB ancestral functions may have been retained in D but lost by SOXN. Alternatively, D may have independently acquired functions distinct from those of SOXN [1].

This work offers an example of how genome-wide studies can link the conservation of major networks to function and assess how pathways have evolved in parallel. Only with a better understanding of functional conservation will we be able to answer the question of what the ancestral brain of bilaterians looked like and what vertebrates and invertebrates still have in common.

## Abbreviations

m: mammalian.

## Competing interests

The authors declare that they have no competing interests.
